# Supplementary material for: High spatial resolution nanoslit SERS for single-molecule nucleobase sensing
Source: Nat Commun. 2018 Apr 30;9:1733. doi: 10.1038/s41467-018-04118-7 (PMC5928045; doi:10.1038/s41467-018-04118-7)
Supplement: Supplementary file 1 — Supplementary Information [file 41467_2018_4118_MOESM1_ESM.docx]

**High spatial resolution nanoslit SERS for single-molecule nucleobase sensing**

Chen et al.


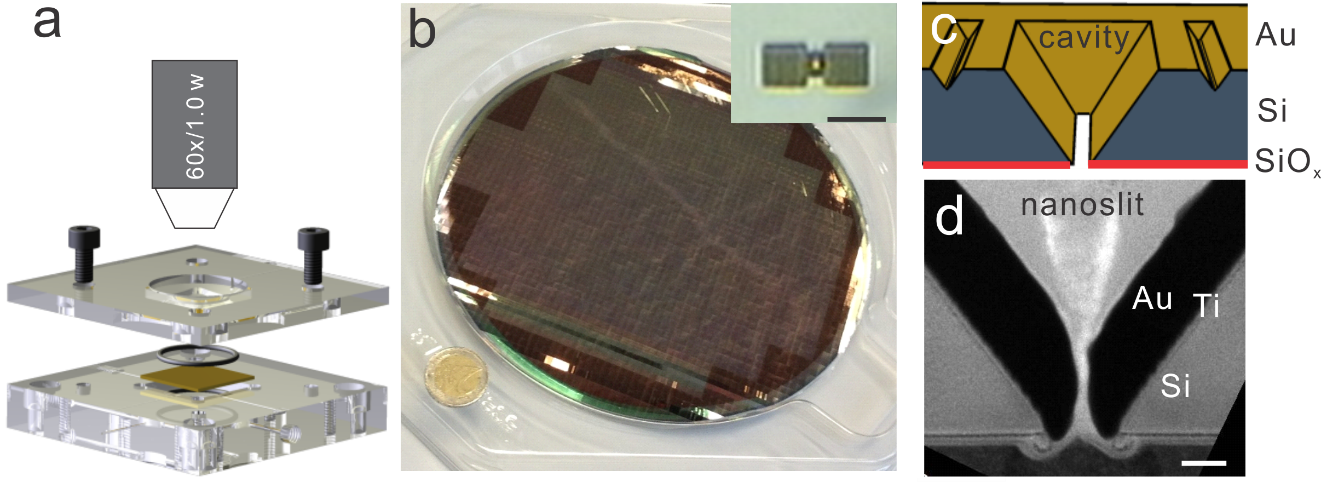


Supplementary Figure 1. Nanoslit-SERS sensor. **a**, Schematic drawing of the flow cell setup. **b,** A 200 mm wafer of nanoslit SERS chips made by our in-house CMOS pilot line. The insert is an optical image of the plasmonic nanoslit device during the measurement. The scale bar is 5 μm. **c,** Schematic drawing of layouts of the nanoslit-cavity structure. **d**, TEM (cross section) image of a zoom-in nanoslit, indicating the sandglass-like cross section of gap, with a neck gap size below 10 nm. The black part is gold, the gray part is Si, and the top bright part is the protection materials (amorphous SiO_2_) for sample preparation by destructive FIB cutting (FEI, Strata 400 STEM at 5 kV). The passivation SiO_2_ at the back was not prepared for this sample and not shown here neither. The scale bar is 50 nm.

**Supplementary Note 1**

# **Spectroscopic library of nucleotides.** Spectra of four nucleotides shown in Fig. 1b are averaged from 100 spectra of each nucleotide. All nucleotides were measured in same experimental configurations and similar nanoslits. For identifying nucleotides, the peak wavenumber and FWHM of the Raman bands are more important than their intensity, as the later one can be influenced by the differentia of nanoslits and optical alignments.

Supplementary Fig. 2a shows same spectra of nucleotides shown in Fig. 1b, but in a broader spectroscopic range of 400 -1800 cm-1. Although the main characteristic Raman bands are within the spectroscopic range of 600 - 900 cm-1, spectroscopic features in other ranges may also be useful for improving the accuracy of identifications. Detailed assignments of SERS bands to vibrational modes of nucleotides are shown in Supplementary Table 1. Figure S2b is the distribution of the peak wavenumber of each nucleotide. Supplementary Fig. 2c is the distribution of the FWHM of Raman bands. We used the local max algorithm to analyze both.


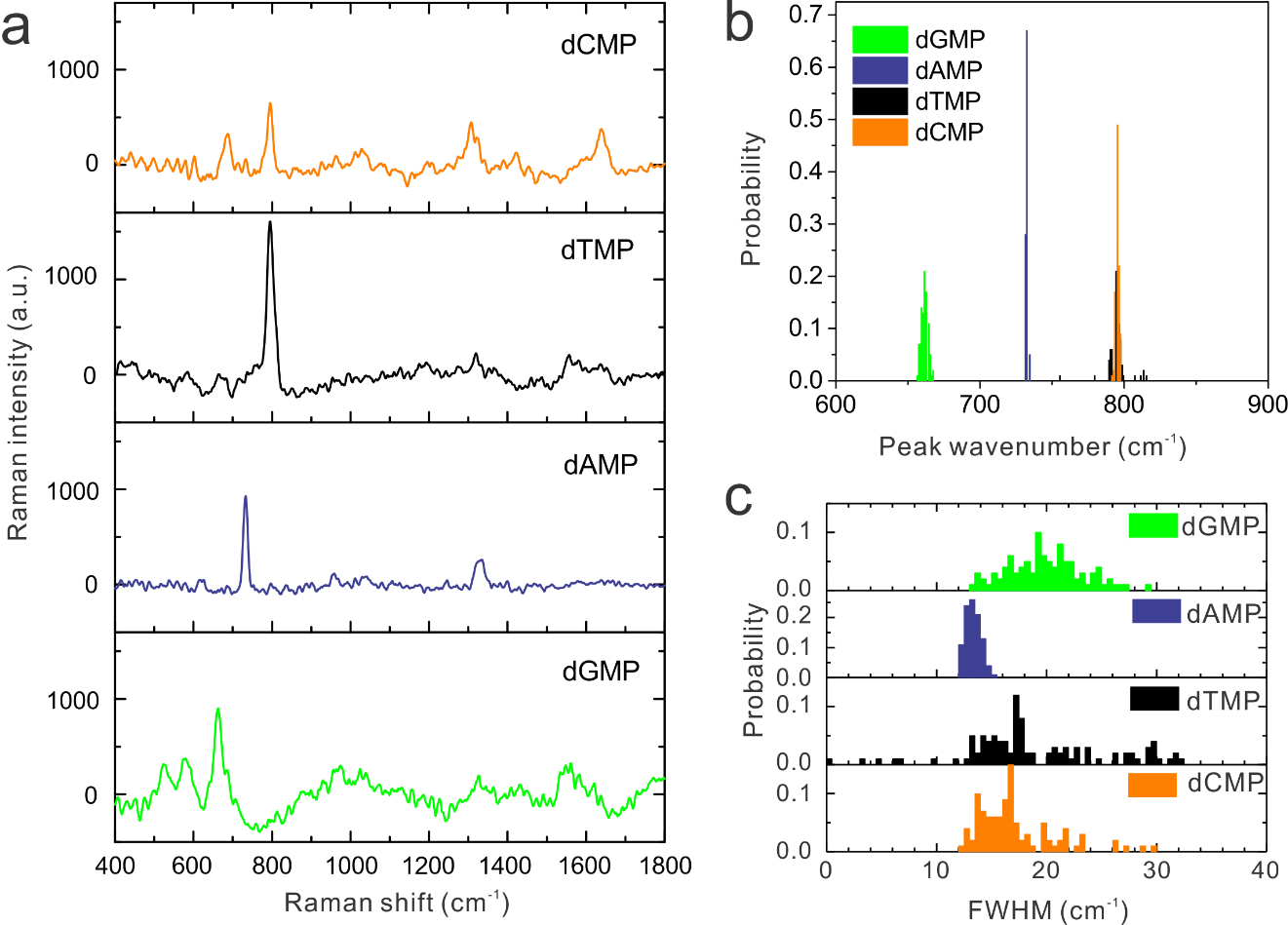


Supplementary Figure 2. Nanoslit SERS of four nucleotides at a high concentration (10^-3^ M in 10 mM KNO_3_ solutions). **a,** averaged spectra of four nucleotides from 100 spectra for each nucleotides. **b,** and **c,** Histograms of the peak wavenumber and the FWHM of main characteristic Raman bands of nucleotides. All spectra were taken at a 8 mW 785 nm excitation, a +0.4 V applied voltage, and a 0.5 s acquisition.

Supplementary Table 1. Assignments of SERS bands to specific vibrational modes of nucleotides

| Raman shift (cm^-1^) | | | | Assignment^1,2^ |
| --- | --- | --- | --- | --- |
| **G** | **A** | **T** | **C** |  |
|  |  |  |  |  |
| 526 m |  |  |  | 6-ring deformation |
| 579 m |  |  |  | 6-ring deformation |
| 663 vs |  |  |  | 6-ring breath, 5-ring deformation, wagging NH2 |
|  |  |  | 686 s | ring deformation |
|  | 733 vs |  |  | ring breath |
|  |  | 793 vs | 796 vs | ring breath |
|  |  |  | 1305 m | ring stretching C-N |
|  |  | 1320 w |  | bending CH3, deformation C6-H |
|  | 1332 m |  |  | stretching C5-N7, N1-C2=N3, C5-C6, bending C2-H |
| 1554 m |  | 1557 w |  | ring stretching |
|  |  |  | 1640 m | stretching C2=O |

Note: vs, very strong; s, strong; m, medium; w, weak.

**Supplementary Note 2**

**Voltage modulated nanoslit SERS.** Similar to other nanopore technologies, we applied a bias voltage cross the membrane to drive molecule motions. A 1 x 10^-3^ M dAMP in 10 mM KNO_3_ solution was used as the analyte sample. In Supplementary Fig. 3a and 3b of voltage-modulation of many-molecule sensing, we can find a clear correlation between the SERS signals and the applied voltages. At +0.5 V, we obtained much stronger Raman bands from dAMP. These bands also blue-shifted 2 - 3 cm^-1^. While at 0 V, we only recorded weak signals of dAMP or even just the background. This correlation can be well repeated in our five-pulse trains (120 s at +0.5 V and 3 s at 0 V). Meanwhile, as shown in Supplementary Fig. 3c and 3d, the voltage-modulation also worked for single-molecule sensing (10^-7^ M adenine in 10 mM KNO_3_). However, the slow diffusion and the small number of analytes disturbed the fluidic response and the intensity of SERS, making the sensing less stable than many-molecule sensing. In addition, we also tested other nucleotides and obtained similar results in. In our other projects, the voltage-modulation for SERS worked well for nanoslits in different sizes too.

We also evaluated a control sample, a 10 mM KNO_3_ blank solution. 1000 spectra were taken from the nanoslit under a 785 nm excitation at 8 mW, a +1 V voltage and a 0.1 s acquisition. As show Supplementary Fig. 4a, we did not observe any SERS features even in the spectrum averaged from these 1000 spectra. In the data analysis, we used the same algorithm for nucleotides. And, we found the distribution of the integrated intensity in the range of 710 - 750 cm^-1^ was centered at 0, and the peak wavenumber appeared randomly. All these mean that only the background noises were involved in the data processing. We further measured this blank sample solution at different applied voltages and only observed background noises. However, when we used a KNO_3_ solution with a much higher concentration of 100 mM, we could observe the normal Raman band of NO_3_^-^ at ~1050 cm^-1^.


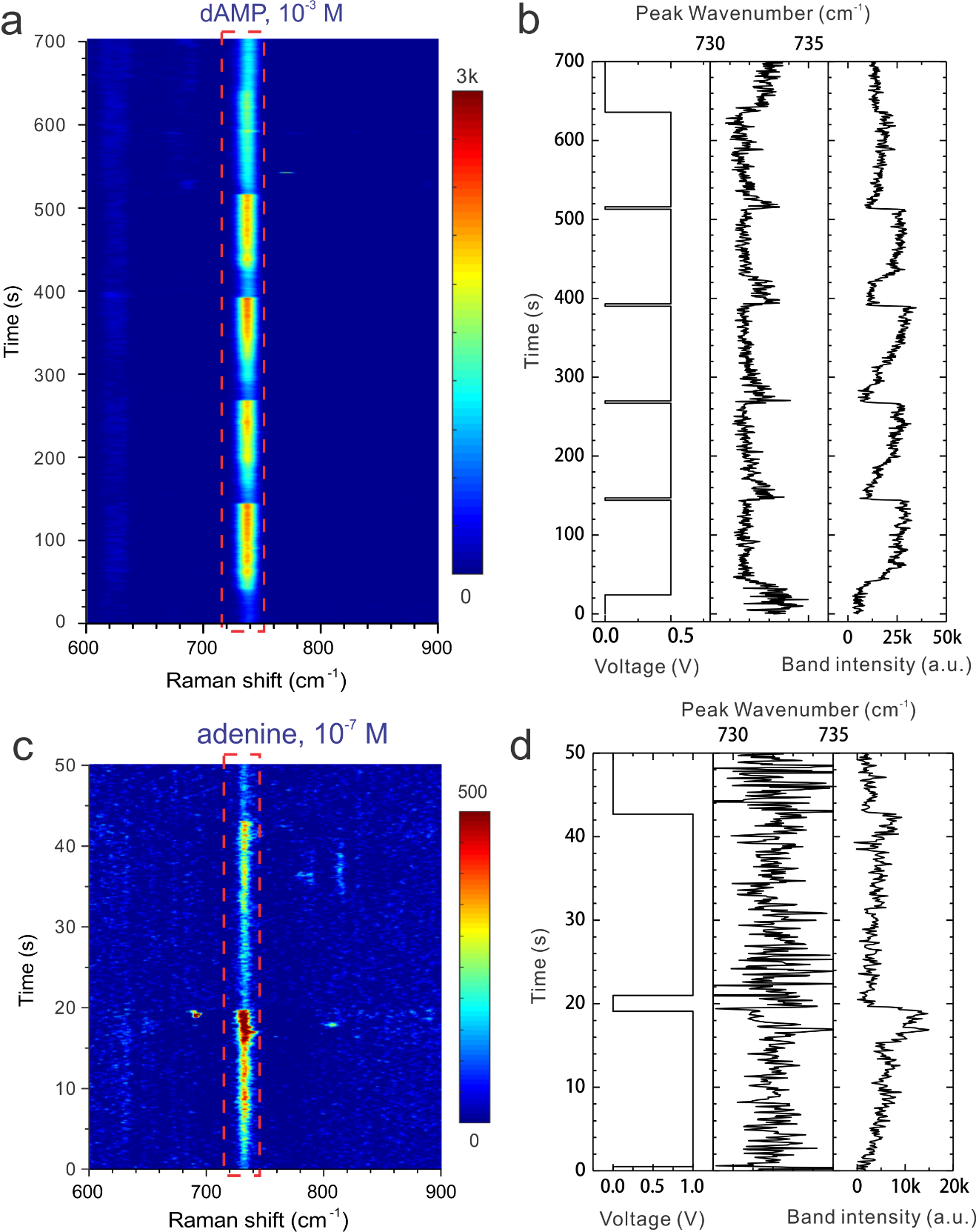


Supplementary Figure 3. Voltage modulation. Many-molecule sensing of 1 x 10^-3^ M dAMP in a 10 mM KNO_3_ solution. **a,** The contour map of SERS from the nanoslit. Five voltage pulses (120 s) at +0.5 V were applied with 3 s intervals at 0 V. **b,** Time traces of the voltage (left), the peak wavenumber (middle) and the integrated intensity (right) of the Raman band of dAMP. Each spectrum was taken at 0.5 s. Single-molecule sensing of 1 x 10^-7^ M adenine in 10 mM KNO_3_. **c,** The contour map of SERS and **d,** time traces of the voltage, the peak wavenumber, and the band intensity of adenine. Each spectrum was taken at 0.1 s.


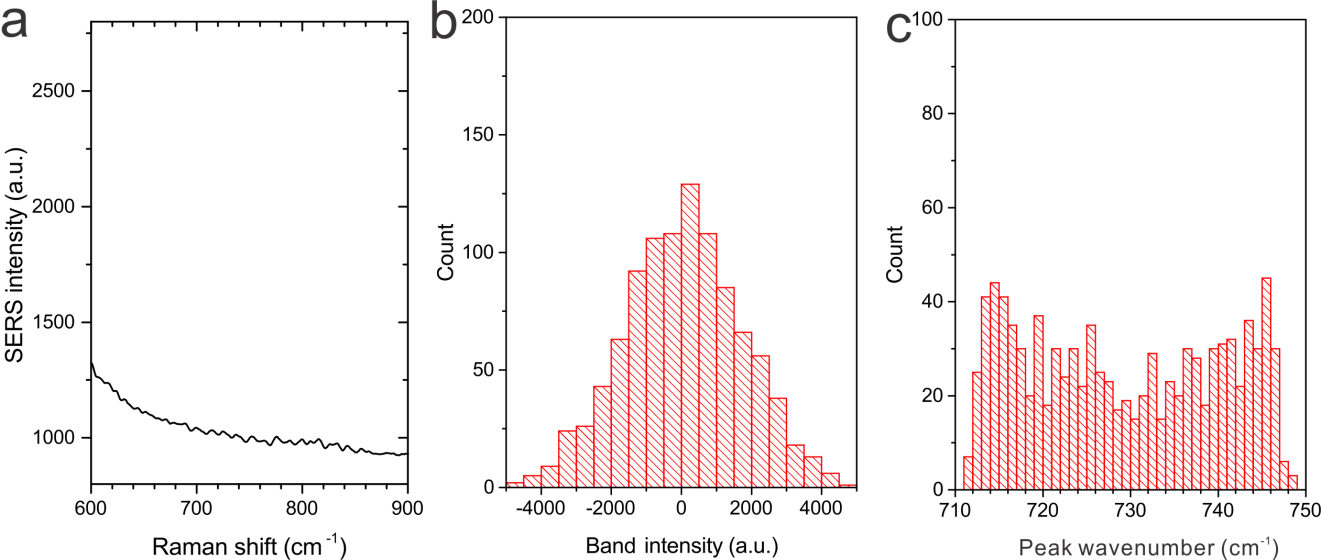


Supplementary Figure 4. A control experiment on a blank sample of a 10 mM KNO_3_ solution. **a,** The averaged spectrum from 1000 spectra taken at 8 mW 785 nm excitation, 0.1 s and +1 V applied voltage. No Raman bands from molecules were observed. **b,** The histogram of the integrated intensity in the spectroscopic range of 710 - 750 cm^-1^. The zero-mean value indicates the recording of only background signals. **c,** The histogram of the peak wavenumber of the bands defined by the Gaussian fitting algorithm. The abnormal flat distribution indicates the observation of background signals.

**Supplementary Note 3**

**Single-molecule BiASERS of adenines.** In BIASERS, we used a pair of ^14^N-adenine (^14^N-A) and ^15^N-adenine (^15^N-A) isotopologues to prove the single-molecule sensitivity of nanoslit SERS. A mixture solution of both adenines at a low concentration of 1 x 10^-7^ M was measured for the single molecule sensing experiment (see Fig. 2). To make the spectroscopic library for these two adenines (see Supplementary Fig. 5 and Supplementary Fig. 6), we measured both ^14^N-A and ^15^N-A solutions at a high concentration of 1 x 10^-3^ M in 20 mM KNO_3_ and 14 mM HNO_3_, respectively. Here, we used nitric acid to dissolve adenines at a high concentration. As a control experiment on many-molecule sensing (see Supplementary Fig. 7), we measured the same mixture solution at a longer acquisition time of 0.5 s. As another control experiment on many-molecule sensing (see Supplementary Fig. 8), we measured a mixture solution at a median concentration of 1 x 10^-5^ M in 10 mM KNO_3_ and 0.5 mM HNO_3_ (remaining from the diluting process). As a reference experiment on single-molecule sensing (see Supplementary Fig. 9), we measured a single-analyte solution of ^14^N-A at a low concentration of 1 x 10^-7^ M in 10 mM KNO_3_. Although the concentrations of the electrolyte (KNO_3_) in solutions were different for many-molecule and single-molecule sensing, we have compared the results of ^14^N-A obtained in both 10 and 20 mM KNO_3_ solutions, and found no influence on the spectroscopic features of resultant Raman bands.

Results of the nanoslit SERS of adenines are shown here from Supplementary Fig. 5 to 9. In each one, the left (a) is a spectrum averaged from 1000 - 2500 spectra, the middle (b) is the distribution of the peak wavenumber and the right (c) is the distribution of the FWHM of Raman bands. In many-molecule sensing of single-analyte solutions, the main characteristic bands of ^14^N-A mostly appeared at ~732 cm^-1^ (see Supplementary Fig. 5) and the bands of ^15^N-A appeared at ~723 cm^-1^ (see Supplementary Fig. 6). Both median FWHMs were larger than 12 cm^-1^. In many-molecule sensing of the mixed-analyte solution (Supplementary Fig. 7 and Supplementary Fig. 8), we found most bands appeared between 725 - 732 cm^-1^. And the median FWHMs were 16 - 18 cm^-1^. In single-molecule sensing of the single-analyte solution of ^14^N-A (10^-7^ M), most bands appeared at ~733 cm^-1^, but the median FWHM was < 10 cm^-1^ (see Supplementary Fig. 9).


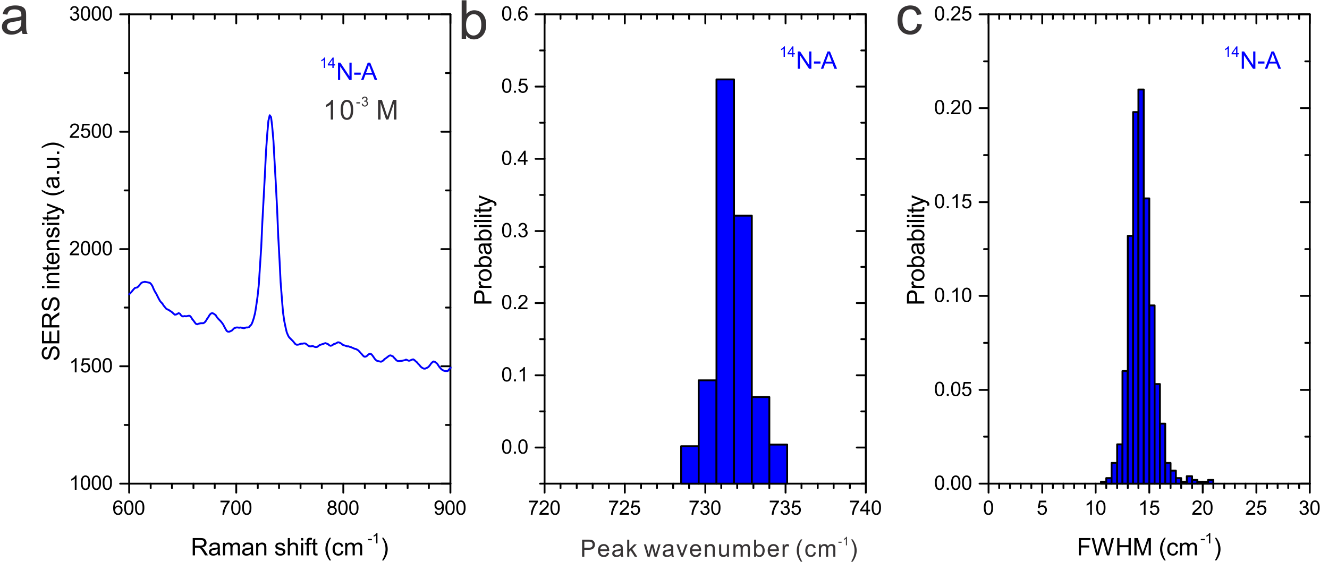


Supplementary Figure 5. Nanoslit SERS of ^14^N-adenine at a high concentration (10^-3^ M in a 20 mM KNO_3_ and 14 mM HNO_3_ solution). **a,** The averaged spectrum from 1000 spectra of adenine taken at 8 mW 785 nm excitation, 0,1 s and +1 V applied voltage. **b,** The histogram of the peak wavenumber of (~732 cm^-1^) of adenine. **c,** The histogram of the FWHM of the band of adenine.


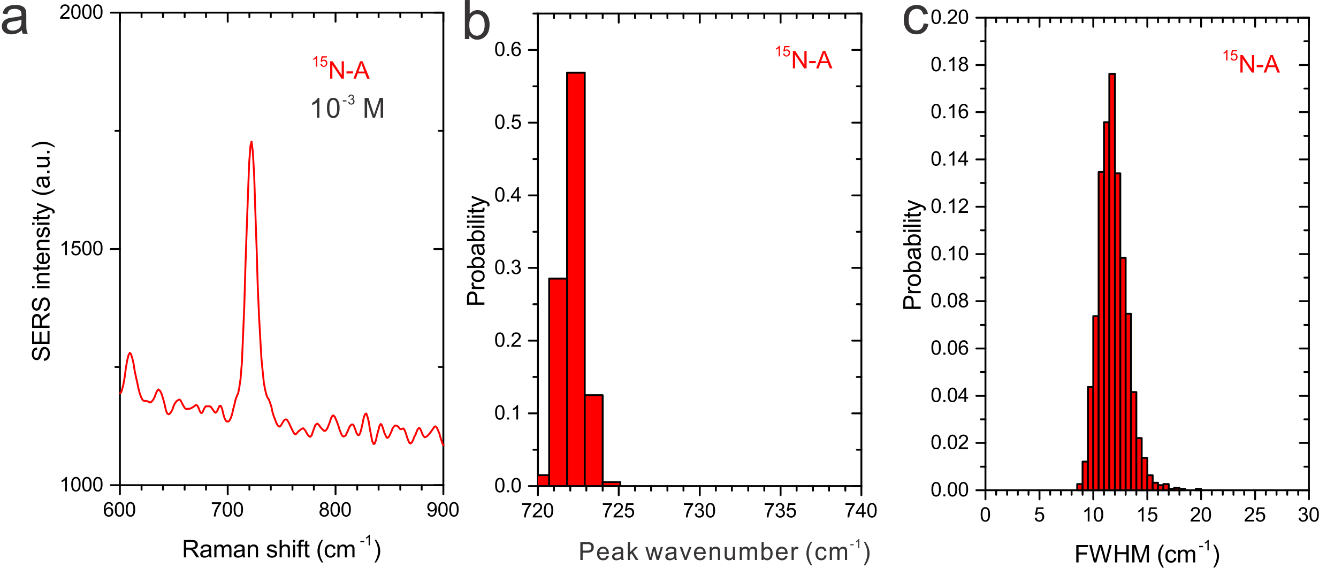


Supplementary Figure 6. Nanoslit SERS of ^15^N-adenine at a high concentration (10^-3^ M in a 20 mM KNO_3_ and 14 mM HNO_3_ solution). **a,** The averaged spectrum from 2000 spectra of ^15^N-adenine taken at 8 mW 785 nm excitation, 0.1 s and +1 V applied voltage. **b,** The histogram of the peak wavenumber (~723 cm^-1^) of adenine. **c,** The histogram of the FWHM of the band of adenine.


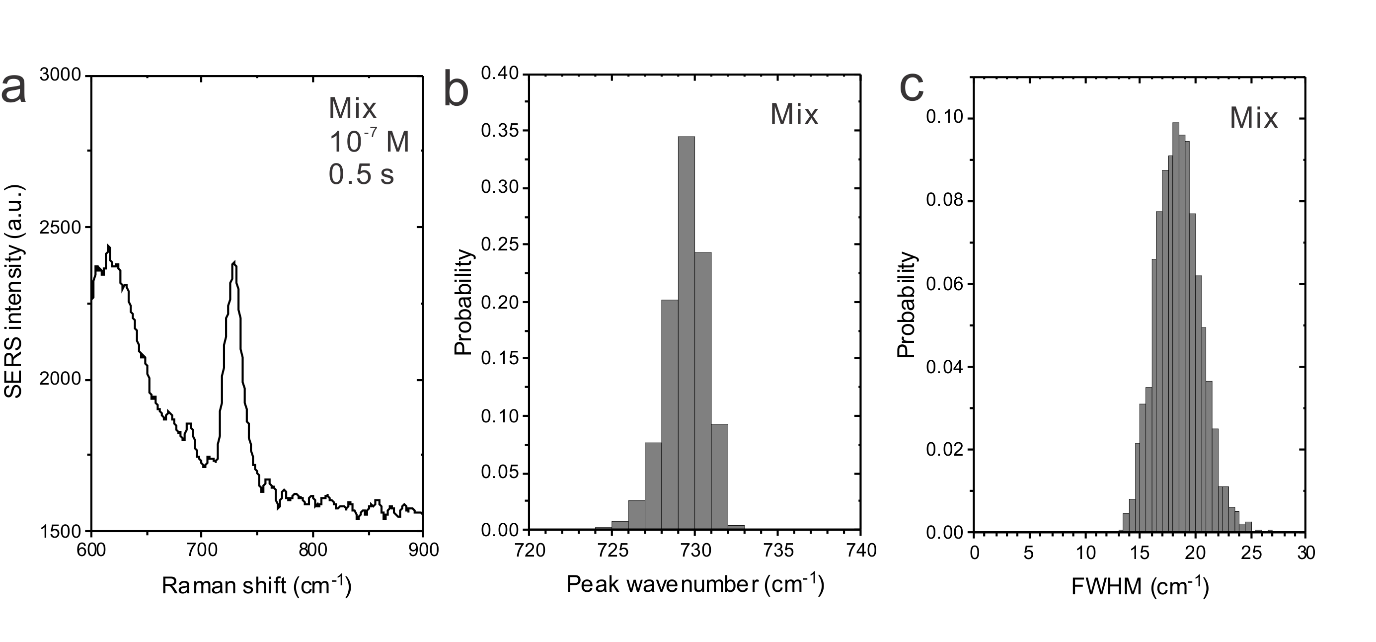


Supplementary Figure 7. Nanoslit SERS of mixed adenine isotopologues (10^-5^ M in a 10 mM KNO_3_ and 0.5 mM HNO_3_ solution) at a slow acquisition time (0.5 s). **a,** The averaged spectrum from 2500 spectra of mixed adenines taken at 8 mW 785 nm excitation, 0.5 s and +0.5 V applied voltage. **b,** The histogram of the peak wavenumber (~730 cm^-1^) of mixed adenines. **c,** The histogram of the FWHM of the band of mixed adenines.


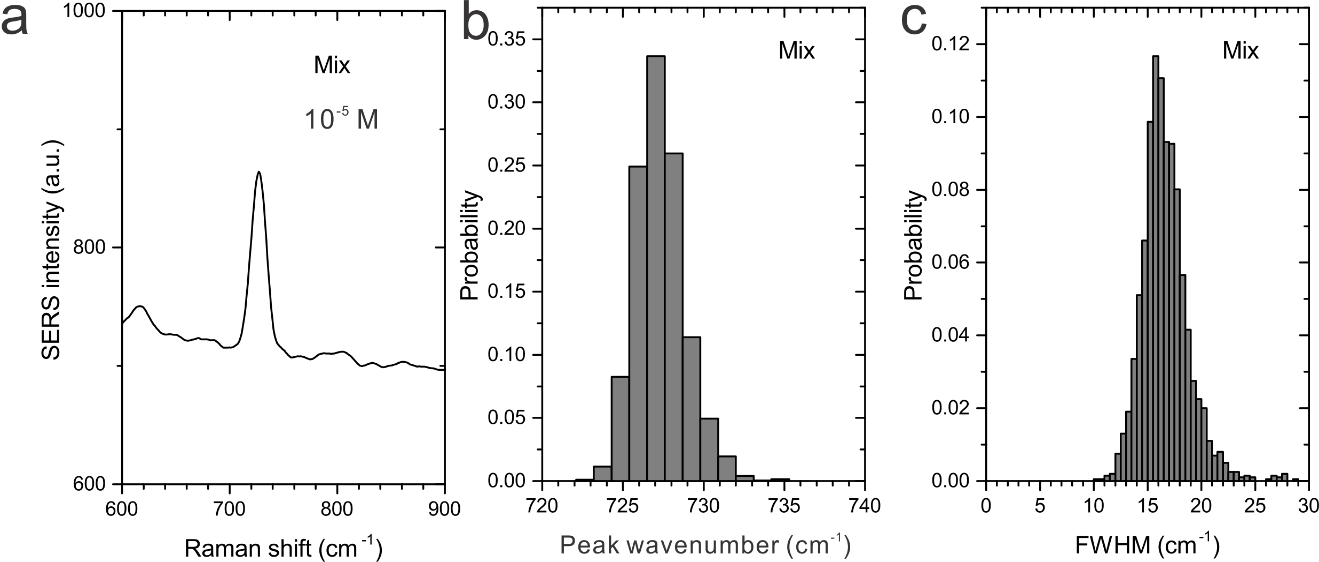


Supplementary Figure 8. Nanoslit SERS of mixed adenine isotopologues of at a quite high concentration of (10^-5^ M in a 10 mM KNO_3_ and 0.5 mM HNO_3_ solution). **a,** The averaged spectrum from 2000 spectra of mixed adenines taken at 8 mW 785 nm excitation, 0.1 s, and +1 V applied voltage. **b,** The histogram of the peak wavenumber (~727 cm^-1^) of mixed adenines. **c,** The histogram of the FWHM of the band of mixed adenines.


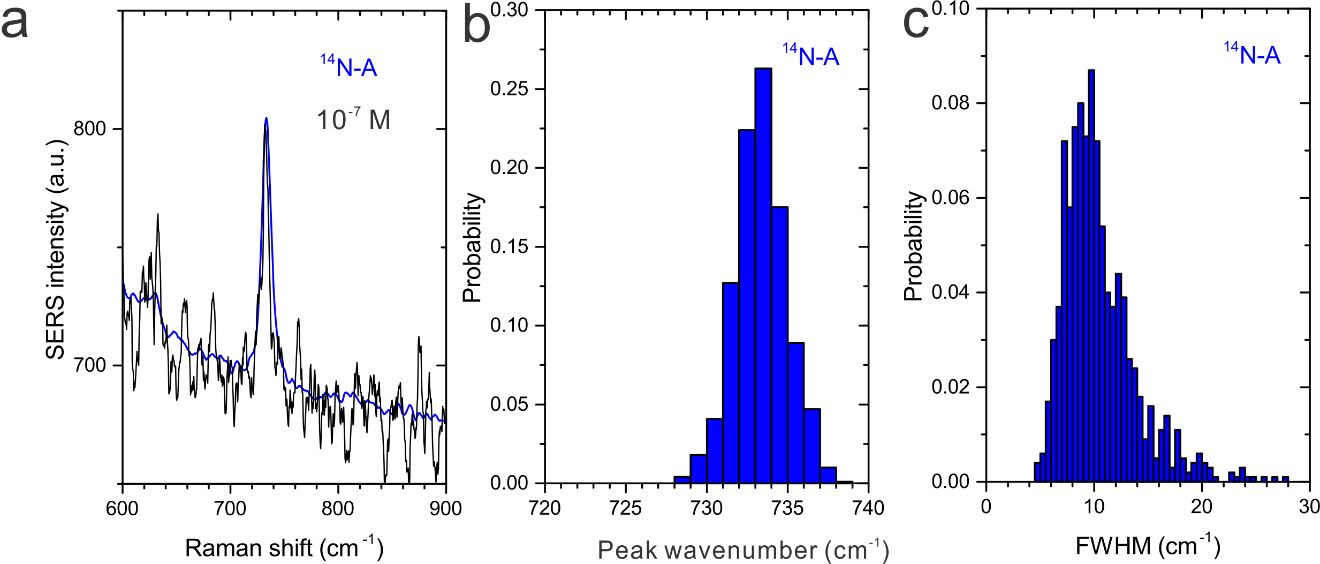


Supplementary Figure 9. Nanoslit SERS of adenine at a low concentration (10^-7^ M in a 10 mM KNO_3_ solution). **a,** The averaged spectrum (blue) from 1000 spectra of adenine and an example spectrum (black) taken at 8 mW 785 nm excitation, 0.1 s and +0.5 V applied voltage. **b,** The histogram of the peak wavenumber (~733 cm^-1^) of adenine. **c,** The histogram of the FWHM of the band of adenine.

Supplementary Table 2. Proportion of different contributions of adenine isotopologues in BiASERS (10^-7^ M and 0.1 s).

| state | single band | events | ratio, % | double-band | events | ratio, % | multiple-band | events | ratio, % |
| --- | --- | --- | --- | --- | --- | --- | --- | --- | --- |
| 1: ^15^N-A dominant | <728 cm^-1^ | 18 | 0.7 | asymmetric, ^15^N-A | 420 | 16.8 | asymmetric, ^15^N-A | 23 | 0.9 |
| 2: ^14^N-A dominant | >732 cm^-1^ | 21 | 0.8 | asymmetric, ^14^N-A | 353 | 14.1 | asymmetric, ^14^N-A | 14 | 0.6 |
| 3: mixed | 728-732 cm^-1^ | 733 | 29.3 | symmetric | 822 | 32.9 | symmetric | 96 | 3.8 |
|  | sum | 772 |  |  | 1595 |  |  | 133 |  |

Note: a symmetric double-band means the ratio of band intensities of two adenines is between 0.5 to 2. Or else, we define it as asymmetric bands, meaning ^15^N-A or ^14^N-A domains the double-band.

**Single-molecule sensing of dGMP.** We further measured another nucleotide, dGMP, at a low concentration of 1 x 10^-7^ M in 10 mM KNO_3_. And we can observe the same narrower FWHM (~ 10 cm^-1^) and the broad distribution of peak wavenumbers of Raman bands (~660 cm^-1^) obtained by nanoslit SERS. The results are shown in Supplementary Fig. 10, further supporting the single-molecule sensitivity of nanoslit SERS.


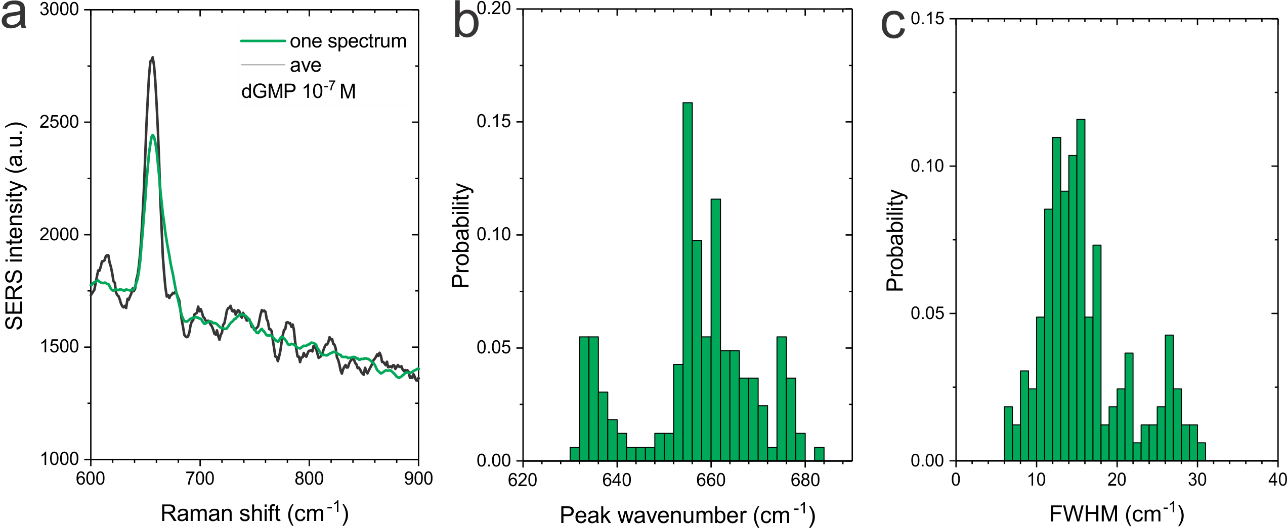


Supplementary Figure 10. Nanoslit SERS of dGMP at a low concentration (10^-7^ M in a 10 mM KNO_3_ solution). **a,** The averaged spectrum (green) from 100 spectra of dGMP and an example spectrum (black) taken at 8 mW 785 nm excitation, 0.2 s and +0.5 V applied voltage. **b,** The histogram of the peak wavenumber (~660 cm^-1^) of dGMP. **c,** The histogram of the FWHM of the band of dGMP.

**Supplementary Note 4**

**Markov diagram.** In single-molecule sensing, the behaviors of ^15^N-A and ^14^N-A should be independent. To validate this, we analyzed the molecular fluctuations in a hidden Markov model, and calculated the probabilities of band transitions between ^15^N-A ,^14^N-A and their mixture. We first should link the observed spectroscopic features to different states of molecules. State 1: in a spectrum, when it only had a single-band < 728 cm^-1^ or a double-band with a larger contribution from ^15^N-A (with a ratio of peak areas > 2), we defined this one as ^15^N-A dominant. State 2: when it only had a single-band > 732 cm^-1^ or the ratio of a double-band is < 0.5, we defined it as ^14^N-A dominant. State 3: when its only single-band was 728 - 732 cm^-1^, or the ratio of a double-band was 0.5 - 2, we defined it as mixed isotopic adenines dominant. The states of multiple-bands were defined in the same way. We then calculated the transition probability between these three states. In the resultant Markov diagram shown in Supplementary Fig. 11, we can see different transition ratios, indicating independent molecular behaviors of ^14^N-A and ^15^N-A. None of the self-transition ratio (e.g. 1 to 1) is close to 100 %. This means that the absorbed adenines inside the sensing region can strongly and stochastically fluctuate, moving in and out of the hot spots frequently and independently.


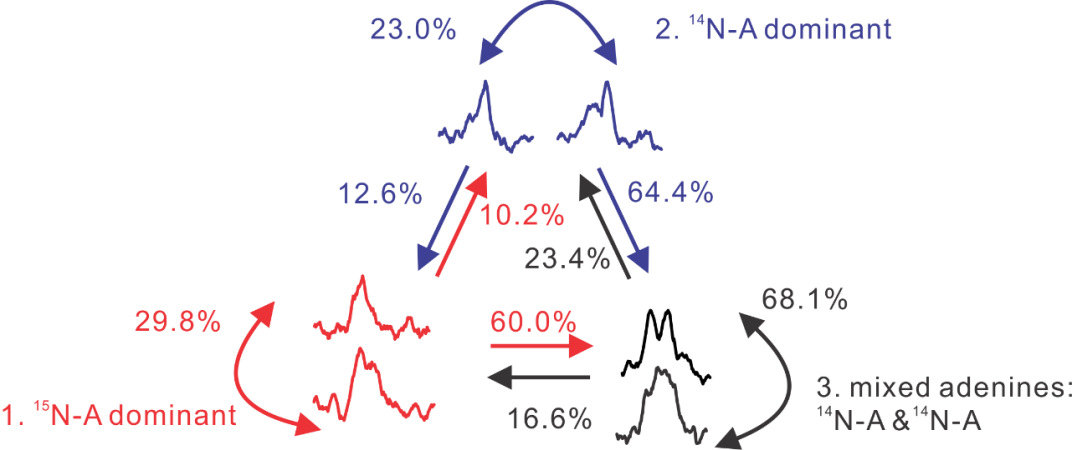


Supplementary Figure 11. The Markov diagram of the band transitions of ^14^N-A dominant, ^15^N-A dominant and their mixed states. Different transition probabilities indicate the independent and strong molecular fluctuations of adenine isotopologues.

**Supplementary Note 5**

**BiASERS of mixted nucleotides.** We have also applied BiASERS for measuring a solution mixed with several of even all four nucleotides. Like in the BiASERS of different adenines, we have tried many different experimental setting for the mixed nucleotides. In most measurements, even at the low concentration of 10^-7^ M, we observed dominating SERS signals from one of the mixed nucleotides. But from a higher concentration solution of mixed nucleotides, sometimes, we did observe independent blinking events of different nucleotides. An example is shown in Supplementary Fig. 12. In the mixed solution of four nucleotides (dGMP, dAMP, dTMP and dCMP) of 5x10^-5^ M in 10 mM KNO_3_ solution, we observed stronger blinking signals (band of 797 cm^-1^) of dCMP and relative stable signals (bands of 651 cm^-1^) of dGMP, thought we did not observe signals of the other two nucleotides. Here, we considered the blinks were dCMP rather than dTMP, as we observed an inverse correlation between bands of 681 cm^-1^ and the bands of 797 cm^-1^, which are both assigned to dCMP. The step-like fluctuations and the narrower FWHM from dCMP can be the evidences for the single-molecule sensing for other nucleotides. However, we would like to emphasis here that using chemically different molecules rather than isotopic molcules can reduce the reproducibility and the probability of asynchronous blinking events.


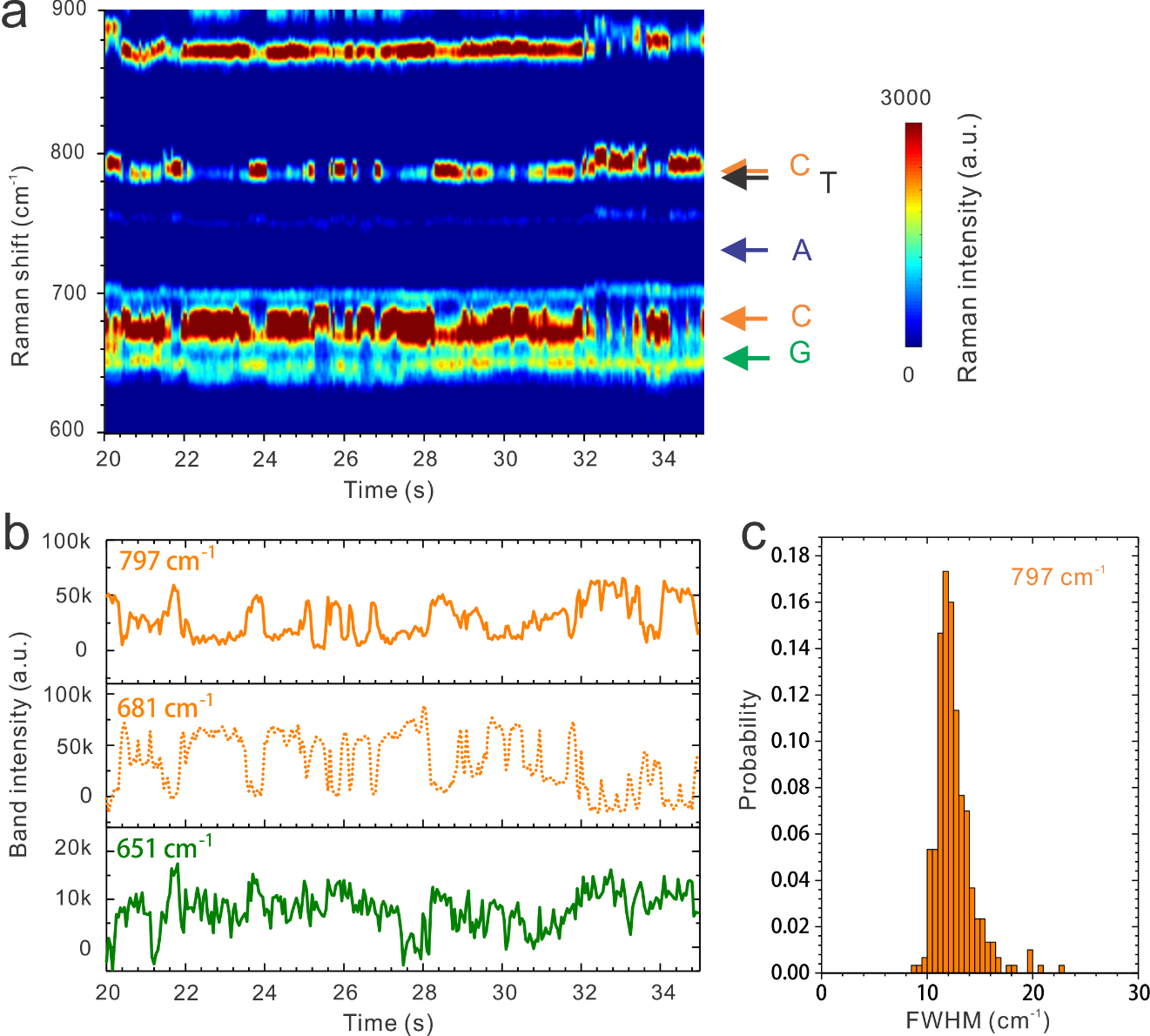


Supplementary Figure 12. Step-like blinking of SERS signals of different nucleotides (5x10^-5^ M of four nucleotides, in 10 mM KNO_3_) taken at 50 ms, exited by a 8 mW, 785 nm laser and a bias voltage of +0.23 V. Blinking characteristic Raman bands of dCMP (797 cm^-1^), dGMP (657 cm^-1^), and the secondary Raman band of dCMP (681 cm^-1^) were clearly observed. **a**, The contour map of SERS and **b,** The intensity fluctuations of the three bands from the mixture solution. Clear step-like blinking of dCMP in the stable-signal of dGMP were recorded. **c,** The histogram of the FWHM of Raman bands (797 cm^-1^) of dCMP. It is narrower than the FWHM of 15 cm^-1^ (Supplementary Fig. 2c) obtained in many-molecule sensing.

**Supplementary Note 6**

**Sub-nanometer resolution for on-strand identification.** For measuring *poly(dA)_48_dCdG* at the single molecule level, we diluted it to 1x 10^-8^ M in 10 mM KNO_3_. For measuring poly(dC)_28_dGdA at the single molecule level, we diluted it to 2x 10^-7^ M in 10 mM KNO_3_. A 785 nm laser at 8 mW was used for excitation. The acquisition time was 0.5 s for the former samples, and 0.05 s for the later samples. The differentia on the experimental configurations was caused by the different affinity of A and C on the gold surface. After applied +0.3 V, we started to observe the SERS signals from samples. Without the bias voltage, we cannot observe SERS signals related to DNA. As shown in Fig. 4b, at the wavenumber near 600 cm^-1^, we also noticed an increasing background signal at 300 – 500 s, which was related to the Raman band of the Au-O vibration. Usually, we considered this as a sign for needs of re-cleaning for the nanoslit device.

We analyzed the FWHM of Raman bands of C, G (blinking events) and A (persistent events). Both the blinking events and persistent events were screened by using an intensity filter at 5000. The background signals can be thus excluded. In Supplementary Fig. 12, the median FWHMs (~10 cm^-1^) of bands of G and C are narrower than that of A. They are comparable to those observed in the single-molecule sensing (see Fig. 2c). While the median FWHM of A (~ 16 cm^-1^) is similar to that observed in the many-molecule sensing (see Supplementary Fig. 2c).


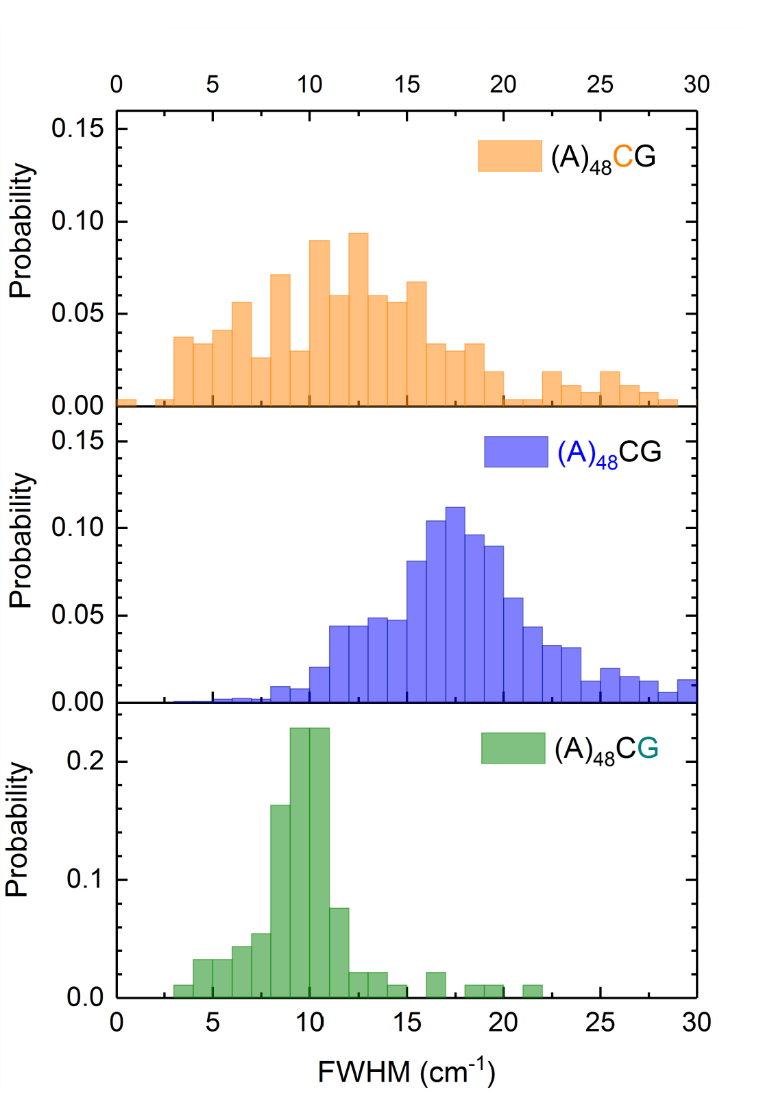


Supplementary Figure 13. The histograms of FWHM of the characteristic Raman bands of C, G and A on the single-stranded DNA oligonucleotides. Narrower FWHM of C and G indicates the single-molecule sensing.

**Supplementary Note 7**

**Replication experiments of same DNA oligonucleotides.** Replication experiments for *poly(dA)_48_dCdG* were carried out and the results of asynchronous blinking of the DNA samples are shown in Supplementary Fig. 13 and Supplementary Fig. 14. Same experimental configuration was used, including the same nanoslit and same sample solution. Applying a bias voltage of +0.3 V, we observed the voltage-modulated adsorption of *poly(dA)_48_dCdG* and the gradually increased SERS signals from A. As shown in Supplementary Fig. 13, several blinking events of C can be recorded. This indicates the transient presence of C in the hot spots, caused by the stochastic fluctuation of the DNA strand. However, no clear events of G were observed. Oppositely, in Supplementary Fig. 14, we can find several blinking events of G, rather than C. The weaker SERS intensity of the bands should be caused by the shorter acquisition time used in this measurement. In addition, we also tried other nanoslits, the persistent signals from longtime adsorption of the dominating bases were not often observed. However, fast asynchronous blinking events of C, G and A were easily observed, which would be related to the high spatial resolution of SERS too.


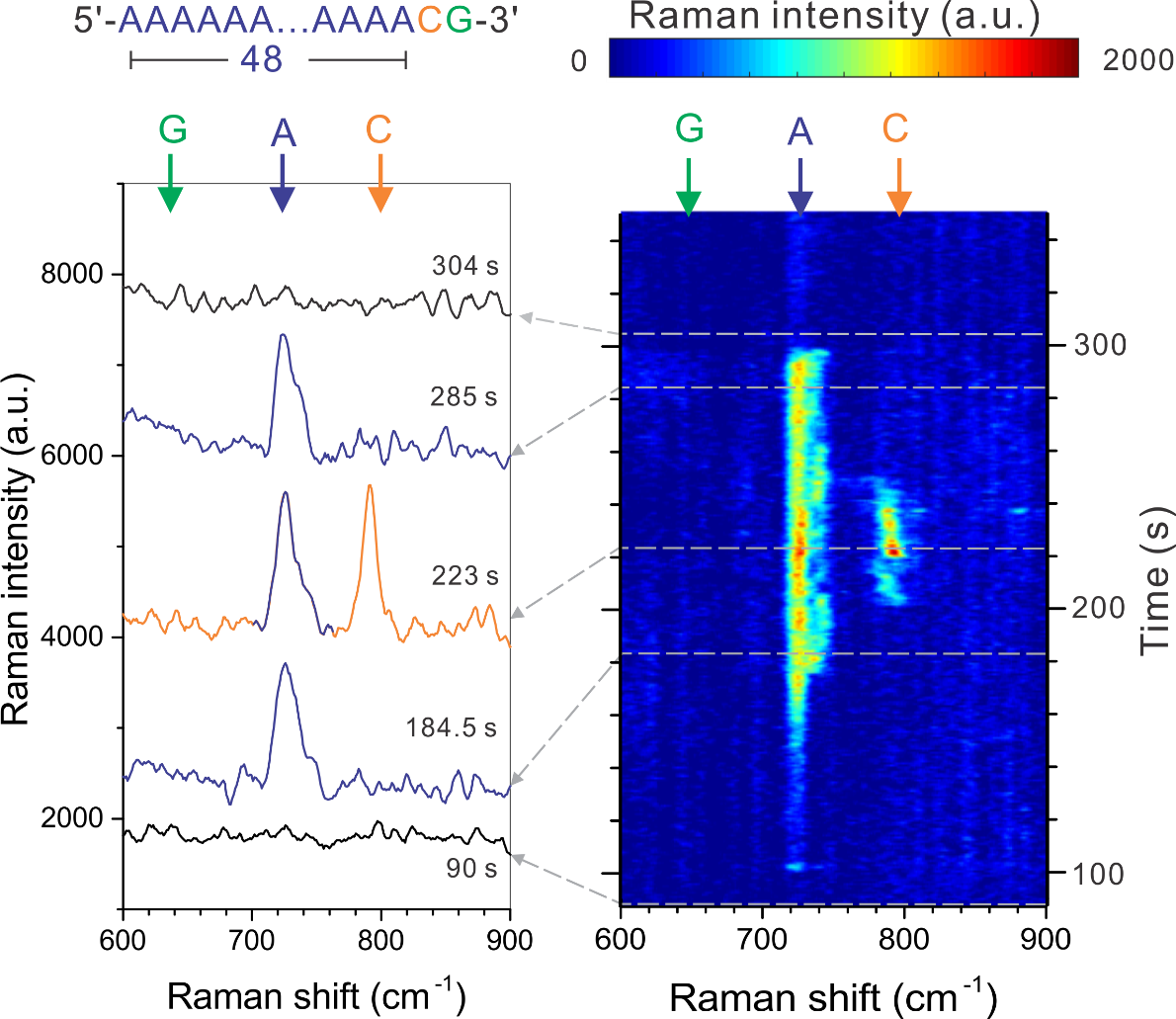


Supplementary Figure 14. Replication for *poly(dA)_48_dCdG* measurement. In the contour map of nanoslit SERS of the ss-DNA (1x10^-8^ M in a 10 mM KNO_3_ solution) and the example spectra (offset), blinks of C in the presence of A were observed again. This measurement was implemented at a 8 mW 785 nm excitation, 0.5 s acquisition for each spectrum, and a voltage of +0.3 V between 150 to 300 s.


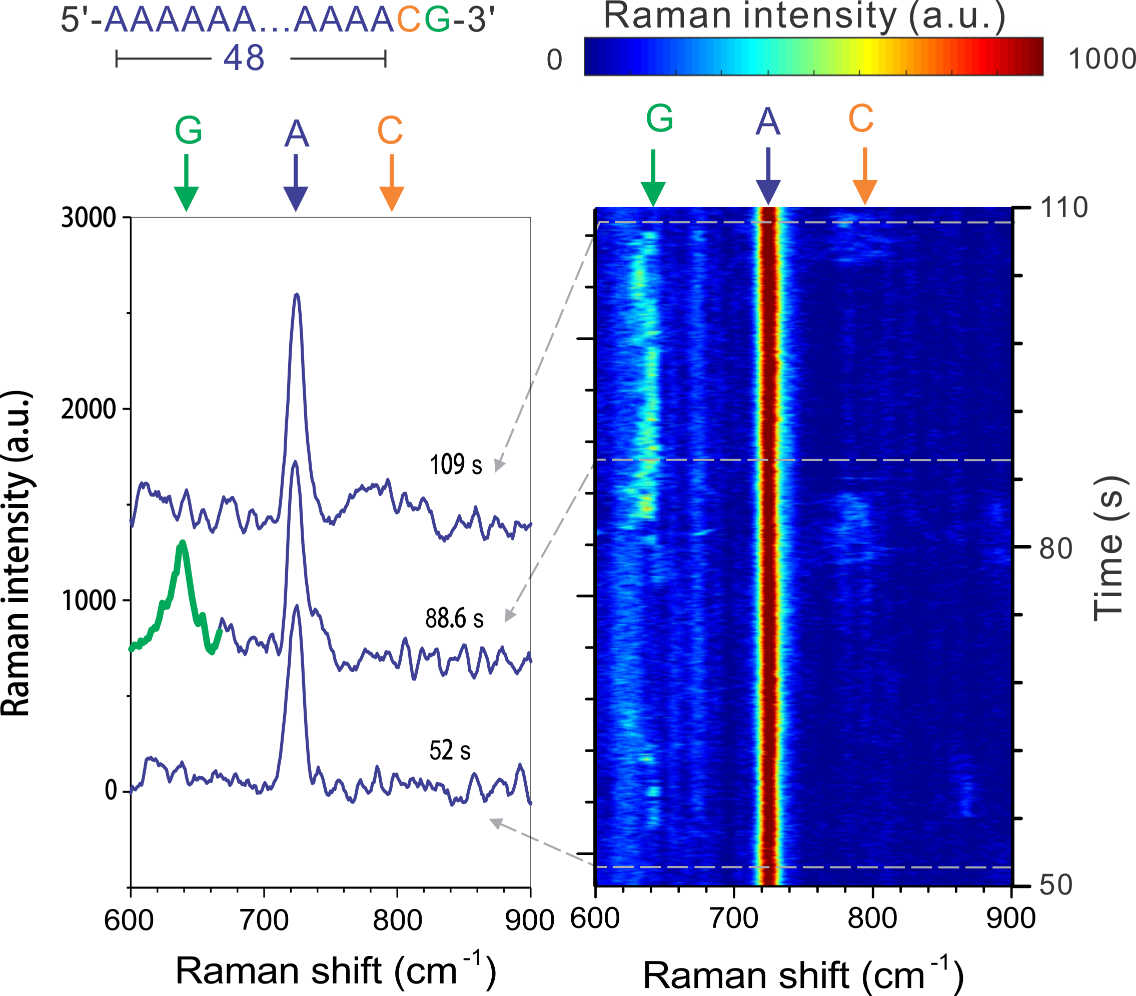


Supplementary Figure 15. Replication for *poly(dA)_48_dCdG* measurement. In the contour map of nanoslit SERS of the ss-DNA (1x10^-8^ M in a 10 mM KNO_3_ solution) and the example spectra (offset), blinks of G in the presence of A were observed again. This measurement was implemented at a 8 mW 785 nm excitation, 0.05 s acquisition for each spectrum, and a voltage of +0.3 V.

**Supplementary Note 8**

**Replication experiment of another DNA oligonucleotides.** To further validate the capability of on-strand identification, we studied another DNA oligonucleotide, *5’-poly(dC)_28_dGdA-3’*. Since the affinity of C is weaker than A on the gold surface,^3^ we can expect an unstable absorption and stronger fluctuation of the strand during real-time measurements. The *poly(dC)_28_dGdA* sample was optimized at a higher concentration of 2 x 10^-7^ M in 10 mM KNO_3_. A same setup configuration was used, except a faster acquisition of 0.05 s to follow the fluctuations. After applying the voltage, we started to observe randomly asynchronous blinking SERS signals of these three bases. As shown in Supplementary Fig. 15, we observe interesting transitions of SERS bands of A, G and C in 102.5 - 103.5 s. It should be noticed that in this trace, the ring deformation mode of C at ~687 cm^-1^ was much more visible than the breathing mode at ~796 cm^-1^, and we assigned this band to C. Obviously, the blinking of A and G happened very fast but asynchronous, while the blinking of C was much more persistent. This fast and asynchronous blinking of nucleobases is like the result discussed in Fig. 4. It clearly supports that its high-spatial resolution is independent on the sequence of the DNA strands, but is an intrinsic property of nanoslit SERS.


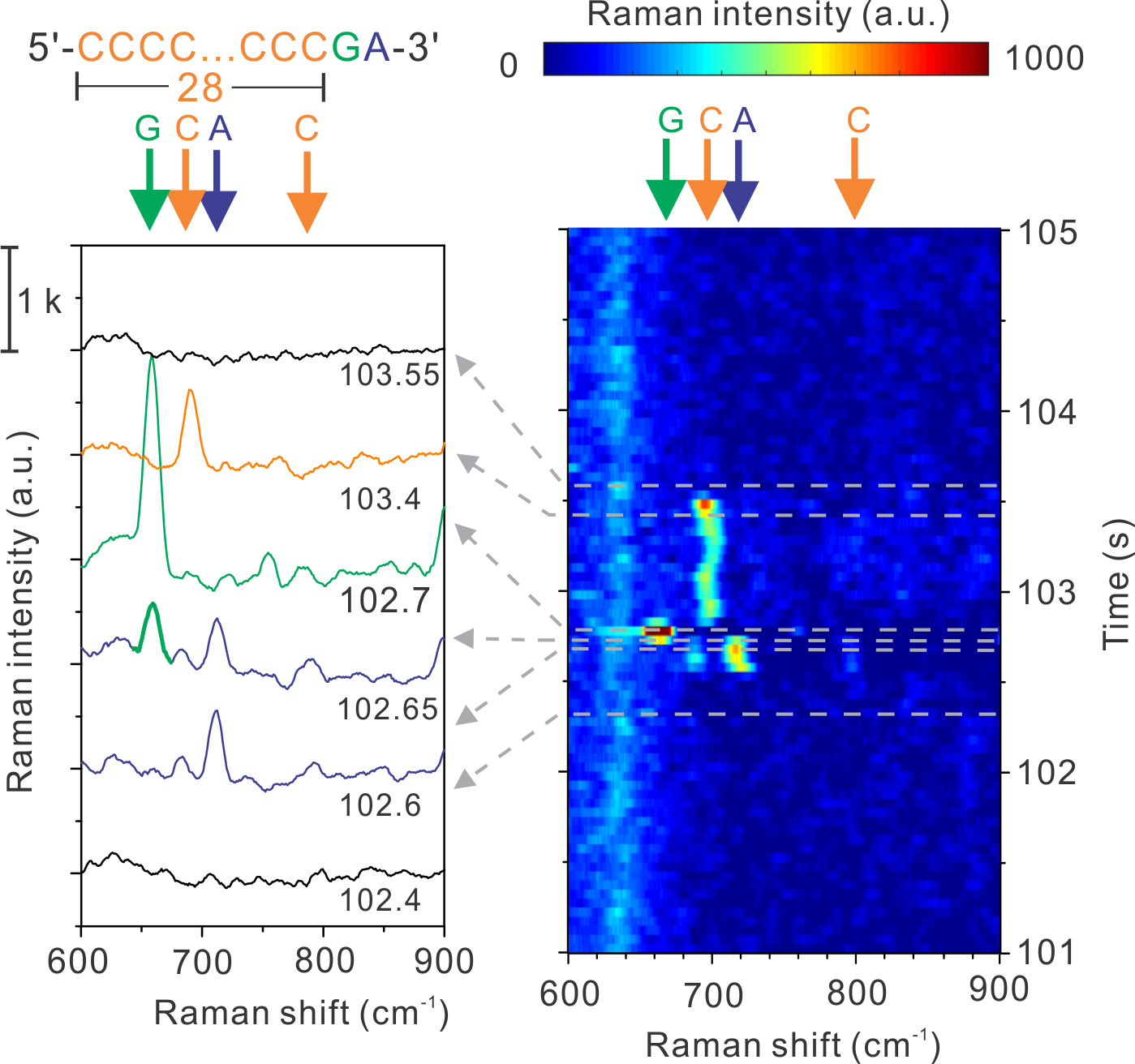


Supplementary Figure 16. Nanoslit SERS for *poly(dC)_28_dGdA*. In the contour map of SERS of the ss-DNA (2 x 10^-7^ M in a 10 mM KNO_3_ solution) and the example spectra, similar asynchronous blinks of A and G in the presence of C were observed again. The voltage of +0.3 V was applied at 0 s. This measurement was implemented at a 785 nm excitation of 8 mW and 0.05 s acquisition for each spectrum.

**Supplementary References**

1. Otto, C., van den Tweel, T. J. J., de Mul, F. F. M. & Greve, J. Surface-enhanced Raman spectroscopy of DNA bases. *J. Raman Spectrosc.* **17,** 289–298 (1986).

2. Madzharova, F., Heiner, Z., Gühlke, M. & Kneipp, J. Surface-Enhanced Hyper Raman Spectra of Adenine, Guanine, Cytosine, Thymine, and Uracil. *J. Phys. Chem. C* **120,** 15415–15423 (2016).

3. Piana, S. & Bilic, A. The nature of the adsorption of nucleobases on the gold [111] surface. *J. Phys. Chem. B* **110,** 23467–23471 (2006).
